# Perceived Barriers Affecting Access to Preventive Dental Services: Application of DEMATEL Method

**DOI:** 10.5812/ircmj.11810

**Published:** 2013-08-05

**Authors:** Mohammadkarim Bahadori, Ramin Ravangard, Baratali Asghari

**Affiliations:** 1 Health Management Research Center, Baqiyatallah University of Medical Sciences, Tehran, IR Iran; 2 School of Management and Medical Information Sciences, Shiraz University of Medical Sciences, Shiraz, IR Iran

**Keywords:** Public Health, Dental Care, Hospital Dental Services

## Abstract

**Background:**

Identifying perceived access barriers to preventive dental services is one of the basic steps to improve the public health.

**Objectives:**

This study aimed to determine the perceived barriers affecting access to preventive dental services in one of Tehran dental clinics in 2012.

**Patients and Methods:**

This research was a cross-sectional descriptive-analytical study conducted in one of Tehran dental clinics in 2012 using decision–making trial and evaluation laboratory (DEMATEL) method. The study sample included all patients (100 patients) who had referred to the endodontic treatment department from 26 - 31 May, 2012. The required data were collected using a questionnaire. Collected data were analyzed using SPSS 18.0 and MATLAB 7.9.0 SPSSS 18.0, as well as, some descriptive and analytical tests including Mean, Standard Deviation (SD), and Independent T- Test.

**Results:**

The five determinants of cost, inconvenience, fear, organization, and patient-dentist relationship were determined as barriers to access to dental services among which the cost and patient-dentist relationship were identified as the first and last priorities with the coordinates (1.4 and 1.4) and (1.25 and -0.65), respectively.

**Conclusions:**

High cost of dental care has led to not referring patients to the clinic. Oral health costs are too high; however insurance organizations have no commitment to support such services. Policymakers, administrators, and insurance organizations have a major role in improving access to dental services. These decision-makers in making their policies can provide the required financial resources, shift the available resources towards preventive care and periodic checkups, and consider providing proper and sufficient places for dental care facilities.

## 1. Background

Preserving, restoring and promoting the public health are the goals of healthcare providers, and one of the major issues in social welfare is equitable provision of health services to the population. These goals can be achieved when the access to health services is a priority. Therefore, access to health services is important for policy makers, managers, service providers, service recipients and insurance organizations. On the other hand, access to health services is one of the human basic and social rights, and providing and increasing it is a duty of the governments (1). Access to health services has also been emphasized in the Iranian constitution so that it has taken this right into account as a universal right and obligated the government to provide public access to the health services through public revenues and public contributions. The meaning of access is to remove economic, systemic, social, cultural and behavioral barriers to use health services (2).

Good access to health services means the provision of "appropriate services in the right place and at the right time"(3) . Access to health services is considered as one of the social justice determinants and a scarce resource which is dependent on the fair distribution of services through a suitable planning (4, 5). Also, access to health resources has been taken into consideration as an indicator of public health (6). Oral diseases are increasingly prevalent, and despite requiring to treatment, only less than 50% of patients refer to the dentists because of the barriers of access to dental care (7). Access to dental care is limited in many developing countries. One of the common problems in the oral health in developing countries has been tooth decay from which, according to the World Health Organization(WHO) estimates, more than 60 to 90% of the children and the majority of the adults suffer (8). Therefore, the WHO has set the Global Oral Goals by 2020 and believes that sufficient financial resources, manpower, equipment and infrastructure are necessary to achieve these goals (9). In Iran, the average visits to the dentists have been 2.95, 6 and 6.41 at the universities type 1, 2 and 3 respectively in 2010 and there has been one dentist for every 11 thousand people (10).

The results of some studies show that access to health care in some cities in Iran is inequitable. Based on the results of a study (2007) conducted in Tehran, about 20% of those in need have been deprived of access to health services (11). Also, the results of Bahadori and colleagues' study (2012) conducted in Golestan in Iran indicate that there is a large gap among the health structural indicators in different cities of this province (12). Based on the results of a study (2012), the decrease in referring patients to the physicians has been due to the increase in out-of-pocket payments (13). Also, according to the Mohammad Pour and colleagues' study results (2002), some factors including lack of awareness, lack of human resources, low income and lack of insurance coverage have resulted in the lack of access of a substantial percentage of the population to the health services(14). Zarrabi and Shaykh Baygloo study results (2011), also, showed that there was a significant difference among the Iran provinces in terms of the existence and number of healthcare facilities and services (15).

About access to dental care, a separation should be made between the patient-related determinants and the determinants associated with service providers (16). Based on the results of some studies, individual, structural, financial and geographical factors have important roles in access to health services (17, 18). Slack-Smith and colleagues (2010) in their qualitative study concluded that patient-dentist relationship, availability of services, fear, cost, and lack of awareness were the important barriers to access to the dental services among the elderly. Also, the suitable economic and social conditions have significant relationships with the number of dental visits (19, 20). In Medicare, there has been a significant relationship between the levels of payments to the dentists and the number of children's dental visits (21). Religious and ethnic disparities, also, affect access to dental care and are associated with individuals' social and economic status, too (22).

In some countries, such as Germany, the Netherlands, Sweden, Canada, England, Denmark, Japan and Australia, private insurance is used for accessing to dental care, and providing these services is committed by health insurance organizations and people are not worried about the payment of fees. In some countries, dentists provide dental services to their patients and treat them in a competitive environment and at market prices (23, 24). Pender has provided a model of population health promotion which has several parts one of which is the patients' perceived barriers reducing patients' commitments to exhibit health promoting behaviors(25). Theoretical framework for this study is taken from the Pender's health promotion model.

## 2. Objectives

This research aimed to study the patients' perceived barriers to access to the preventive dental services in one of Tehran dental clinics in 2012.

## 3. Patients and Methods

This research was a cross-sectional descriptive-analytical study conducted in one of Tehran city dental clinics in 2012 using decision–making trial and evaluation laboratory (DEMATEL). The study sample included all patients (100 patients) who had referred to the endodontic treatment department from 26 - 31 May, 2012. The formula for calculation of sample size was n= (Z_ɑ/2_ + Z_β_)^2^ / d^2^ (26). This formula was determined using the findings of previous studies, as well as considering power analysis and the assumption of normality (assuming α = 0.05, β = 0.1, d = ɛ/σ = 0.32). The opinions of all available experts (using consensus method) were studied. However, studied patients were selected using systematic random sampling method and included the patient had referred to the endodontic treatment department in morning and evening shifts in during one week. The inclusion criteria for this study were those patients who had a felt need or one of the prognoses of sticking the dental floss between the teeth, sensitivity to the cold water and the sweet foods, and swollen gums in some areas before coming to the endodontic treatment department, however, they had not referred to the dental clinic for various reasons before root canal treatment. Therefore, because all of the referred patients to the endodontic treatment department had at least one of the inclusion criteria, no one was excluded from the study.

The required data were collected using a researcher made-questionnaire and available literature reviews. In order to identify the barriers to access to dental services, all available resources were reviewed. Then, the deduced barriers were discussed and categorized in a focus group discussion. Afterwards, a questionnaire was designed using the categorized barriers and sent to 30 experts specializing in dental care and health services management who were faculty members of Tehran University of Medical Sciences to express their viewpoints on the identified determinants and assess its validity. This questionnaire had five closed questions and one open question. The closed questions were rated using a Likert scale. The open question was designed so that they were able to express their viewpoints on the mentioned determinants.

When the barriers were confirmed, another researcher- made questionnaire was designed to collect needed data from patients. This questionnaire consisted of two parts. The first part included the patients' demographic data and the second one included the barriers that had been compared with pairwise. A 5-point scale was used to assess the determinants and determine the priority of each determinant over all other ones (9 = extremely important, 7= Very important, 5 = Important, 3 = Slightly important, and 1 = Equal importance). The questionnaire validity was done through content validity done by the dental care and health services management experts from the country. In order to do so, the experts were asked to comment on the content of the designed questionnaire and its items in the written format. The reliability of the questionnaire was approved by Cronbach Alpha Coefficient (α = 0.80).

Kolmogorov-Smirnov test was used to specify the normality of studied data. So the normality of studied data was confirmed.

For data collection, the third author had attended in the endodontic treatment department of clinic for a week and identified those patients who had inclusion criteria and could enter the study. The study [CH/7018/11] was approved by the medical research ethics committee of the Baqiyatallah University of Medical Sciences in June 2011. Participation in this study was voluntary for the patients and it was ensured that their information would be completely confidential. Therefore, required data were collected, recorded and analyzed anonymously in such a way that the studied patients could not be identified. The patients were fully informed about the purpose and nature of the study. Then, informed consent was obtained from all patients for participating in this study. It should be noted that the questionnaire did not include the patients' emotional, psychological and behavioral aspects. The collected data were analyzed using SPSS 18.0 and MATLAB 7.9.0, as well as, some descriptive and analytical tests including Mean, Standard Deviation (SD), and Independent T- Test. DEMATEL is an operations research method which is used to structuring the factors which affects a phenomenon. This method is one of the modeling methods and categorizes the studied factors into two groups: affecting factors and affected ones(27, 28).

## 4. Results

Based on the findings of this study, 33 variables were identified which were categorized into five determinants including cost, fear, inconvenience, patient-provider communication and organization (Table 1). Results showed that the mean score for each of the determinants had acquired more than 75 percent of the score. In other words, all the experts agreed with the proposed barriers affecting access to dental services (P = 0.0001) (Table 2). Based on the findings of this study, 65 patients (65%) were male and 58 patients (58%) were married. The mean age of studied patients was 31. 91 ± 8.55, and the most of them (38 patients, 38%) were in the 20 - 30 age group. Most of the studied patients (58 people, 58%) had diploma and all of them were insured (Table 3). 

**Figure 1.**
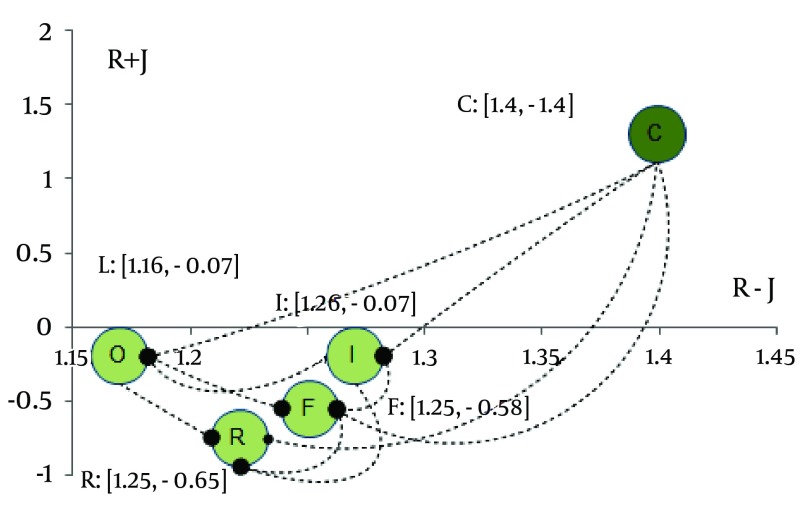
The prioritization of and relationship between access barriers to preventive dental services, (C, Cost; F, Fear; I, Inconvenience; O, Organization; R, Relationship)

As shown in Figure 1, the factors were visually divided into groups according to whether R-J was positive or negative. So the cause group with positive R-J value included cost, and other determinants including organization, inconvenience, fear and the patient-dentist relationship located in the effect group since they had negative R-J. Combining Table 4 and Figure 1, we can analyze each system factor and discuses factors' impact on the whole system, thus the barriers of preventive dental services can be figured out. The results showed that cost was a certain affecting determinant which influenced the access to dental services and was placed in the affecting factors group. Also, organization, inconvenience, fear and the patient-dentist relationship were affected and placed in the affected factors group (Table 4). 

On the other hand, cost and patient-dentist relationship were identified as the first and last priorities with the coordinates (1.4 and 1.4) and (1.25 and -0.65), respectively. Table 4 shows that the degree of influential impact of cost, the only casual factor, was 1.4. It indicates that cost has remarkable impact on other factors, and that improvement of cost can lead to amelioration of preventive dental services. To sum up, cost was an important factor and should be received much more attention in preventive dental services. 

**Table 1.** The Recognized Variables and Barriers and Their Categorizations

Determinants (Barriers)	Variables
**Cost**	High costs of dental services
	Travel costs
	Low levels of dental insurance coverage
	Opportunity cost
	Low income
**Inconvenience**	Annoying methods and procedures used by the provider
	Need to plan ahead to make an appointment with the dentists in the clinic
	Long distance from home to the clinic
	Complex and prolonged dental treatments
	Lack of service providers' attention to simple treatments
**Fear**	Fear of the dentist
	Fear of the injection of the anesthetic materials into the teeth
	Fear of encountering serious problems after treatment
	Patients' unwillingness to be examined
	Patients' unwillingness to respond to the providers' questions
	Fear of dentist's diagnosis
**Patient-Provider Relationship**	Patient's poor speech
	Culture and poor attitude of patients to receive dental care
	Lack of service providers' attention to the patient's problems
	Impatient service providers
	Failure to respond to the patients' questions
	Not giving enough information to the patients
	Lack of clinical response to the patient s' questions via telephone
**Organization**	Having no choice of service providers by the service recipients
	Lack of modern equipment and supplies
	Poor physical appearance
	Long waiting times
	Inappropriate waiting rooms
	Lack of proper parking space
	Inappropriate location of clinic
	Not clean clinic
	The low quality of services
	Lack of trust in the service provider

**Table 2.** Experts' Viewpoints on the Perceived Access Barriers to the Dental Services

The Perceived Barriers	Respondents Comments					Mean	SD	P Value
	Completely agree	Agree	No comment	Disagree	Completely Disagree			
**Cost**	19	8	3	0	0	4.53	0.68	0.001
**Patient-Provider Relationship**	14	4	2	0	0	4.40	0.62	
**Organization**	14	13	3	0	0	4.10	0.99	
**Inconvenience**	18	9	2	0	1	4.43	0.64	
**Fear**	20	10	0	0	0	4.66	0.44	

**Table 3.** The Demographic Data of Studied Patients

Characteristics	N (%)
**Sex**	
Male	65 (65%)
Female	35 (35%)
**Age Groups**	
20 >	8 (8%)
20-30	38 (38%)
31-40	36 (36%)
41-50	18 (18%)
> 50	0(0%)
**Marriage Status**	
Married	58 (58%)
Single	42 (42%)
**Education Level**	
Diploma	58 (58%)
Bachelor's Degree	30 (30%)
Master's Degree	9 (9%)
Ph.D.	3 (3%)
**Insurance Coverage Status**	
Insured	100 (100%)
Uninsured	0 (0%)

**Table 4.** The Hierarchy of Affecting and Affected Access Barriers to Preventive Dental Services

Determinants	Cost	Fear	Incontinence	Location	Relationship	R	J	R + J	R - J
**Cost**	0	0.3881	0.3515	0.3438	0.3166	1.4	0	1.4	1.4
**Fear**	0	0.0187	0.2139	0.0449	0.0571	0.3346	0.9204	1.25	-0.58
**Inconvenience**	0	0.0891	0.0187	0.2139	0.2717	0.5934	0.6732	1.26	-0.07
**Organization**	0	0.2106	0.0442	0.0093	0.2818	0.5459	0.6213	1.16	-0.07
**Relationship**	0	0.2139	0.0449	0.0094	0.0210	0.2802	0.9392	1.21	-0.65

## 5. Discussion

Access to the health services and disparities in access to dental services have been considered by the World Health Organization Commission on Social Determinants of Health, and the health ministries of countries have been asked to give priority to these issues(29). Paying attention to the access programs can lead to promote oral health(30). In this study, the researchers tried to examine the access barriers to dental services systematically to achieve a greater understanding of these determinants using a conceptual model. Also, the priorities of these determinants were determined based on their relationships and the intensity of their influences. Based on this study results, "cost" as an affecting determinant was identified as the first priority and distress, fear and the patient-provider relationship, respectively, received the next priorities.

In a study (2007) conducted in Iran which aimed to represent the experience of accessing to the health services, some components such as low cost of healthcare services, the appropriate time, place and communication skills had been considered by the patients referred to the health centers. Also, Poor economic and social status could lead to patients refer to a physician when their disease had reached its acute phase(31). The results of a study in Bulgaria (2009) showed that the barriers to access to health services were poverty, cultural, geographical and managerial barriers (32). Fitzpatrick et al in their study of the barriers to access to physician concluded that the most important ones were lack of attention to patients' concerns as one of the variables of patient-service provider relationship, the variables associated with the inconvenience, as well as, financial components. They, also, showed that the physical and psychological barriers played a prominent role in access to health services(33). Al-Shammari et al (2007) in a study in Kuwait concluded that fear, bad habits and false beliefs were the major access barriers (34)which their findings on fear confirm the results of the present study. Although fear is a barrier, it is one of the components which took the last priorities. The results of some studies have shown that fear has a direct relationship with not referring to the dentist(35, 36).

Based on the results of a study (2011), organizational factors were the most important barriers in access to health care and financial factors were the least important ones from the viewpoints of the employees of an urban health center(17) which are different from the results of the present study. The reason for this difference can be found in the type of services provided because the types of services provided in a dental clinic are different from those offered in an urban health center. In other words, the services offered in an urban center are free. However, those services provided in a dental clinic not only are not free but also have a heavy cost. In a study conducted in Malaysia (2011), the quality of services, as one of the structural determinants, was reviewed. This study results showed that there was a fundamental difference between patients' expectations and their perceptions, and service providers should pay more attention to this difference (37).

The results of Telleen et al (2012) and Wellstood and colleagues' (2006) studies showed that organizational barriers were one of the access barriers (1, 38). "Location" of service providers is the other determinant affecting the access to health care. In other words, if these facilities are not in the right places, inappropriate places will be one of the access barriers (38, 39). In the present study, location is considered as one of the organizational variables. Improving access to healthcare requires the optimal establishment of health care facilities(5). If service providers do not find the right location, it will increase the patients' costs and the travel time for receiving the needed care. Therefore, this determinant has been considered by many planners so that they try various models and methods out, especially mathematical models, to establish the health service facilities in the proper places and whereby they can remove one of the access barriers. Toregas, Eaton, Cheng and their colleagues have reviewed this determinant in their studies (40-42).

Another determinant affecting healthcare functions and increasing the access to healthcare is appropriate and effective relationship(43). The improper patient-service provider relationship is one of the barriers to access to health services. Good relationship is a key factor. If the patient cannot ask the provider its needs or if service provider does not give sufficient information to the patient, the relationship will be flawed and will prevent proper access. The results of Mohammad Pour and colleagues' study (2002) showed that 22% of people had not referred to the physician in a health center because of the lack of confidence in him/her (14). In the present study, patients had identified the inappropriate patient-provider relationship as the last priority which is different from the Mohammad Pour and colleagues' study results. In other words, this determinant is the most affected factor in the model of access barriers to dental care.

Despite the WHO emphasis on the patients' fair financial contribution to health costs, a large part of the costs is still paid by the patients. Although it is a strategy for funding and reducing the cost of insurance organizations, it is considered as a major barrier to population access to health care (44-47)Wharam et al (2007) in their study concluded that increasing the contribution of patients led to a reduction in necessary visits in the hospital emergency department(48) . The results of the Newhouse and colleagues' (1981)study also showed that the patient's contribution to the costs of health care had reduced the average use of the healthcare(49) . In a study (2006) conducted in Tehran, there was not any significant relationship between insurance status and the use of dental care (50). In the present study, despite the fact that patients were insured, the results showed that being insured was not enough and the level of dental care coverage is more important.

Based on the Bailit and D'Adamo's study results (2012), there are some limitations for improving equity which one of the most important one is the per capita income. High per capita income has the major role in reducing access to dental services. This has resulted in a lot of programs have not been become operational (51).Junqueira et al believe that to promote people's health, it is necessary to take substantial measures in the field of social exclusions(52) . In the present study, "cost" was known as the most affecting determinant. Patients considered "cost" as a factor that had prevented them from accessing to dental care in the dental clinics. Curtis et al (2007) in their study examined some of the barriers to access to dental services and concluded that the indirect costs such as travel costs in remote areas had effect on access to dental care and was considered as an important barrier (53). Among Canadian adults, also, travel costs and having a low income had been considered as two access barriers(54). Some countries attempt to increase the equitable access to health services through insurance coverage(1).

The results of a study (2011) conducted in India showed that the cost and the felt need influenced the number of dental visits; however, the distance from the dental clinic had no significant effect(7). While in the present study, despite the fact that patients felt the need, they would not go to the dentist until their disease reached its acute phase including needing for root canal treatment. In another study, 93% of patients had gone to the dentist only when they had faced with a problem, and their unfelt need was a major barrier to access to the dental services(55).

Since the people's conditions and characteristics affect their access to health services, the policies on distribution of health services for the population should be fair and equitable(56). Today, in many countries, the private sector plays an important role in providing health services. Supporting the private sector by insurance organizations increases the population utilization of and access to health care(57). Cost as an important access barrier to dental care can affect the other influential barriers. High cost of dental care has led to not referring patients to the clinic. Oral health costs are too high; however insurance organizations have no commitment to support such services. Policymakers, administrators, and insurance organizations have a major role in improving access to dental services. These decision-makers in making their policies can provide the required financial resources, shift the available resources towards preventive care and periodic checkups, and consider providing proper and sufficient places for dental care facilities. Establishing dental clinics in the appropriate places so that patients have easy and equitable access to them, using management tools to reduce waiting times in busy clinics, and constructing the new clinics because of the increased demand are important ways.

The strength of this study was its accuracy of assessment and measurement. One of the decision making technique and pairwise comparisons were used in this study and the studied patients compared the considered determinants reciprocally by which the measurement accuracy was increased. Also, the causal relationships between the determinants were specified using this method. The present study had some limitations. In this study, we only studied the determinants; however, the variables associated with each determinant were not reviewed because of patients' fatigue and tiredness and therefore, lowered patients' accuracy in their responses. Also, this study was conducted on patients whose diseases had reached an acute and critical stage, while selecting the study sample from households could increase the study accuracy.
